# NMR-Metabolic Methodology in the Study of GM Foods

**DOI:** 10.3390/nu20100001

**Published:** 2010-01-13

**Authors:** Anatoly P. Sobolev, Donatella Capitani, Donato Giannino, Chiara Nicolodi, Giulio Testone, Flavio Santoro, Giovanna Frugis, Maria A. Iannelli, Autar K. Mattoo, Elvino Brosio, Raffaella Gianferri, Irene D’Amico, Luisa Mannina

**Affiliations:** 1 Institute of Chemical Methodologies, Magnetic Resonance Laboratory “Annalaura Segre”, CNR, Via Salaria km 29.300, 00015 Monterotondo, Rome, Italy; Email: donatella.capitani@imc.cnr.it (D.C.); flavio.santoro@hotmail.it (F.S.); 2 Institute of Biology and Agricultural Biotechnology, CNR, Via Salaria km 29.300, 00015 Monterotondo, Rome, Italy; Email: donato.giannino@ibba.cnr.it (D.G.); chiara.nicolodi@mlib.cnr.it (C.N.); frugis@ibba.cnr.it (G.F.); mariaadelaide.iannelli@ibba.cnr.it (M.A.I.); giulio.testone@ibba.cnr.it (G.T.); 3 Henry A. Wallace Beltsville Agricultural Research Center, United States Department of Agriculture, Agricultural Research Service, Sustainable Agricultural Systems Laboratory, Beltsville, Maryland 20705-2350, USA; Email: Autar.Mattoo@ARS.USDA.GOV; 4 Chemistry Department, University of Rome “Sapienza”, p.le Aldo Moro, 5 00185, Rome, Italy; Email: elvino.brosio@uniroma1.it (E.B.); raffaella.gianferri@uniroma1.it (R.G.); 5 STAAM Department, University of Molise, Via De Sanctis, 86100 Campobasso, Italy; Email: irene.damico@unimol.it; 6 Faculty of Pharmacy, University of Rome “Sapienza”, p.le Aldo Moro, 5 00185, Rome, Italy

**Keywords:** NMR metabolic profiling, genetically modified food, transgenic lettuce

## Abstract

The ^1^H-NMR methodology used in the study of genetically modified (GM) foods is discussed. Transgenic lettuce (*Lactuca sativa* cv "Luxor") over-expressing the *ArabidopsisKNAT1* gene is presented as a case study. Twenty-two water-soluble metabolites (amino acids, organic acids, sugars) present in leaves of conventional and GM lettuce were monitored by NMR and quantified at two developmental stages. The NMR spectra did not reveal any difference in metabolite composition between the GM lettuce and the wild type counterpart. Statistical analyses of metabolite variables highlighted metabolism variation as a function of leaf development as well as the transgene. A main effect of the transgene was in altering sugar metabolism.

## Abbreviations

Ala, alanine; Asn, asparagine; Asp, aspartic acid; COSY, correlated spectroscopy; Caff-Tartar, monocaffeoyl-tartaric acid; Fruc, fructose; GABA, γ-aminobutyrate; β-Gluc, β-glucose; Gln, glutamine; Glu, glutamic acid; HSQC, heteronuclear single quantum coherence; Ile, isoleucine; NMR, nuclear magnetic resonance; NOESY, nuclear Overhauser and exchange spectroscopy; PCA, principal component analysis; Sucr, sucrose; Thr, threonine; TOCSY, total correlation spectroscopy; TSPA, 3-(trimethylsilyl)propionic-2,2,3,3-*d*_4_ acid sodium salt; Val, valine.

## 1. Introduction

The introduction of engineered DNA sequences into the plant genome to confer new properties and improve the plant is an active research field. Different complementary approaches, such as genomics, proteomics and metabolomics are useful tools for the study of transgenic organisms, each technique specifically contributing to the overall understanding of the system [1]. In the recent years, the study of metabolic phenotypes as the basis for discriminating between comestible plants of different genotypes has become a topic of considerable scientific interest. Moreover, the system-wide metabolic analysis is becoming a useful tool for functional genomics [2]. NMR spectroscopy as a high-throughput analytical method is a powerful tool to study the metabolic profiles of foods of either animal or crop origin [3,4,5,6,7]. NMR-based identification and quantification of metabolites requires minimal preparation and handling, and no derivatization. The NMR-metabolomic methodology has been applied to study a number of genetically engineered (GM) plant species including lettuce (*Lactuca sativa* cv. "Cortina") expressing the *asparagine synthetase A* gene [8], tomato (*Solanum lycopersicum* cv "Ohio 8245") expressing the yeast *SAMdc* gene [9], maize expressing the *Cry1A*(b) gene [10], several transgenic wheat lines [11], and six transgenic lines of pea (*Pisum sativum*) [12]. 

The essential points and specific recommendations for the effective application of NMR methodology in the study of foods are highlighted and discussed here. A novel case study analyzed is the transgenic lettuce line, which over-expresses the *A. thalianaKNAT1 *homeobox gene, under the control of pea plastocyanin promoter (*PetE)*. Traits and biological aspects of *PetE:KNAT1* lettuce lines have been previously characterized [13,14]. Briefly, GM leaves exhibit vascular net modifications (mid vein shortening and hyper-branching), lamina margin modifications (lobbing and fringing), compact curly butter-heads and flowering anticipation compared to control wild type. Here, the NMR spectroscopic methodology was used to identify and profile the levels of variation in water-soluble metabolites in transgenic and wild type lines at two stages of leaf development.

## 2. Results and Discussion

As is the case of most chemical methodologies, unambiguous application of NMR in metabolomics of transgenic organisms requires that prior to any analysis particular attention is given to production, collection and preparation of biological samples. The extraction and/or measurement procedures must not alter the metabolites or their composition, and the data must be reproducible.

### 2.1. Sample Production

Ideally, GM crops and the conventional counterparts should share an identical genetic background so as to address metabolic differences specifically to the transgene action. In strictly self-fecundating crops such as lettuce the homozygosis grade is very high and all marketed varieties are (virtually) highly inbred [15]. In this context, the performance of a given (stable homozygous) GM cultivar is compared to the same non GM cultivar that provided material for the transformation. Moreover, a set of controls are useful to assess whether the metabolic changes are a consequence of the transformation process (e.g., events of T-DNA insertion and position) or the (desired/unexpected) transgene functioning (see section 3.1)

A tight control of environmental conditions (light intensity, photoperiod, temperature, humidity, *etc.*) and conditions of cultivation (soil composition, watering, fertilization, *etc.*) can minimize the exogenous effects on plant metabolite variation. Towards that aim, the glasshouse growing system offers a good solution, since it allows similar, if not identical, conditions for the production of modified and the wild type crops (e.g., lettuce) [8]. The metabolic changes due to variation in environment or the cultivation method can be monitored by NMR to reveal the corresponding metabolic patterns and the interactions of environmental and genetic factors at the level of metabolome [16].

### 2.2. Sampling

Sampling procedures should consider diurnal cycles of metabolism, particularly in situations where these may alter the metabolite content. A limited number of cases where NMR was used for the metabolic study of plant development [8,9,17] showed that each developmental program of plant organs (leaves, fruits, *etc.*) is reflected in unique metabolic changes, processes that are developmentally regulated. Consequently, the GM and control plant tissue to be analyzed must be sampled from the plants at the same or comparable developmental stage. A sufficient number of samples for every combination of factors (genetic, environmental, *etc.*) should be analyzed to obtain statistically significant results. It is understood that the larger the variability of metabolic content, the higher is the number of samples required for the analysis. The number of GM and control samples should be comparable to avoid bias in the statistical treatment of data. Proper storage of plant tissues is required to prevent the metabolite decomposition; low temperature storage (−20°C or even lower) and lyophilization can minimize storage-based decomposition.

### 2.3. Sample Preparation for the NMR Analysis

Simple methods for extraction of water-soluble and lipo-soluble metabolites need to be applied [6,18]. The use of deuterated water for a direct extraction of metabolites from lyophilized tissues is a simple method to avoid any intermediate steps (for example, extraction by H_2_O followed by freeze-drying). The major part of water-soluble metabolites has acid-base properties (organic acids, amino acids, phenols, amines, *etc.*). The simultaneous presence of protonated and deprotonated forms of metabolites and the labile equilibrium between them makes an NMR spectrum pH-dependent. An increment or decrement of pH value as low as 0.1 unit can give rise to an upfield or downfield shift of NMR signals of 0.02 ppm or even higher which corresponds to 12 Hz at 600 MHz (5-10 times the line width). The strong pH-dependence of NMR signals makes the comparison of aqueous extracts with different pHs difficult and precludes the application of automatic or semi-automatic routines for intensity reading. This problem can be overcome using buffered solutions. The concentration of the buffer should be sufficient to assure the minimum pH variation whenever a new kind of plant sample is to be analyzed. For example, a 400 mM phosphate buffer was sufficient for buffering the aqueous solution of metabolites from 25 mg of lyophilized tomato powder [5,9]. On a different note, a relevant loss of sensitivity was observed when a high buffer concentration was used, particularly with cryogenically cooled probes [19]. The phosphate buffer is a suitable one for NMR applications because it has only exchangeable protons that do not produce additional ^1^H signals. For the same reason, many organic acids and bases can not be used for buffering except the completely deuterated ones. 

According to our data, the NMR signals of several organic acids (citric, malic) capable of chelating metals are broadened probably due to paramagnetic cations (Fe^3+^, Mn^2+^) present in plant tissue extracts. These cations can be chelated by EDTA. In fact, EDTA addition into neutral solutions gives rise to a sharpening of the ^1^H-NMR signals for citric and malic acids. 

The stability of metabolites during extraction procedures and NMR measurement should also be taken into account. For example, the endogenous enzymatic activity may induce the hydrolysis of sucrose to fructose and glucose. 

### 2.4. Acquisition of NMR Spectra

As mentioned above, the advantage of NMR application in metabolite analysis is the ability to identify a wide range of compounds directly from mixtures of metabolites. The important characteristic in this context is the dynamic range of an NMR spectrometer. The larger the dynamic range, the higher the concentration range and the number of metabolites that can be simultaneously analyzed in an experiment. In the case of modern high-field NMR spectrometers, the dynamic range is higher than 10^5^ [20]. Another important advantage of the NMR spectroscopy relative to other techniques is the ability to determine the concentration of any metabolite using the same internal standard. Concerning the NMR measurements, several important factors are: sufficient time for relaxation (that can be assured by using longer relaxation delay and/or lower pulse duration), signal to noise ratio sufficient to detect minor components, and proper solvent signal suppression to assure the optimal dynamic range. 

### 2.5. NMR - Metabolic Application: Characterization of Transgenic and Conventional Lettuce

**Figure 1:**
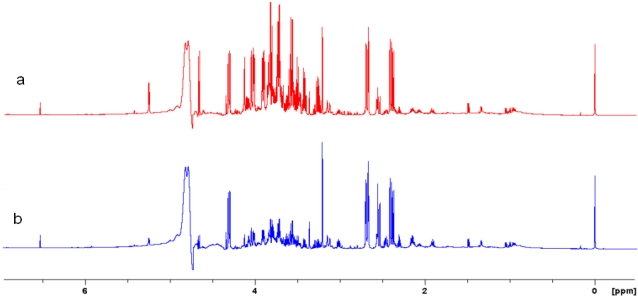
^1^H-NMR spectra of water soluble metabolites from leaves (head stage) of (a) conventional "Luxor" lettuce and (b) transgenic *PetE:KNAT1 *lettuce.

As an example of NMR metabolic profiling of GM foods, a study of transgenic lettuce (*Lactuca sativa* cv "Luxor") over-expressing the *KNAT1* gene from *Arabidopsis* is reported. ^1^H-NMR spectra of water soluble extracts of control and GM lettuce leaves are shown in Figure 1a and Figure 1b, respectively. The ^1^H spectral assignment was obtained using 2D experiments and as previously reported [6,8]. These spectra were conservative, as previously observed for both conventional and transgenic "Cortina" [8]. No novel or unusual signals were observed when the spectra of aqueous extracts from *PetE:KNAT1 *leaves and conventional "Luxor" counterparts were compared. The position of peaks in ppm was identical in both genotypes, nevertheless intensity differences in some peaks were clearly apparent (Figure 1).

**Figure 2:**
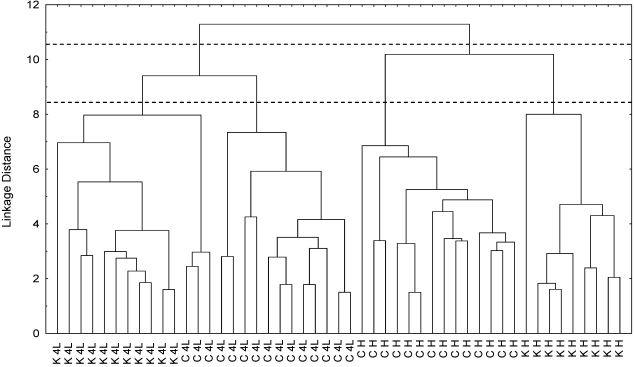
TCA dendrogram applied to GM (K) and control (C) lettuce cv “Luxor” samples at different growth stages. K4L, GM samples at 4^th^ leaf stage; KH, GM samples at the head stage; C4L, control samples at 4^th^ leaf stage; CH, control samples at the head stage.

The intensity of major water soluble metabolites seen in the ^1^H-NMR spectra was quantified for both GM and control leaves from “Luxor” plants at two growth stages. The tree cluster analysis was used to classify samples of transgenic (*PetE:KNAT1*) and conventional lettuce without any *a priori* hypothesis (see Figure 2). 

By making a cut-off in the dendrogram at the highest level, all the samples separated into two clusters: the first one includes all *PetE:KNAT1 *and control samples at the 4^th^ leaf stage, while the second one includes GM and wild type samples at the head stage. A clear effect of the development is present. If the cut-off is made at a lower level, other sub-clusters are observable in which samples are grouped according to the genotype. Thus, the effect of two factors can be observed: the natural leaf development and the genotype. The leaf development program appears to have the most significant impact on the metabolome. 

**Figure 3:**
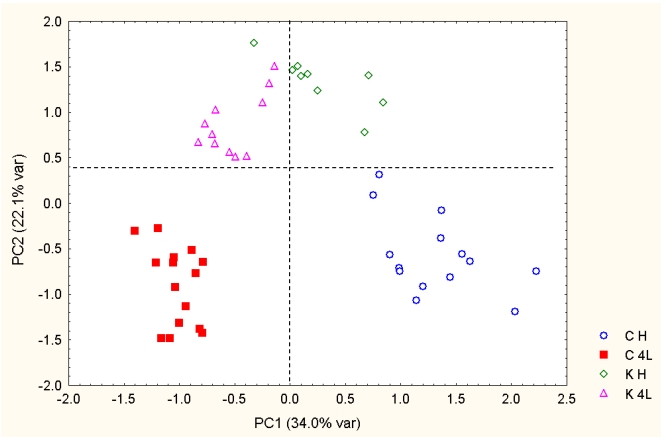
PCA map of lettuce sample scores. C, conventional samples; K, *PetE:KNAT1* samples. K4L, GM samples at 4^th^ leaf stage; KH, GM samples at the head stage; C4L, control samples at 4^th^ leaf stage; CH, control samples at the head stage.

The natural grouping of lettuce samples can be also explored using principal component analysis (PCA). The PCA map performed on 49 samples is shown in Figure 3; the combined PC1 and PC2 explain 56.1% of variance. The samples along PC1 are grouped according to the developmental stage: the head stage lies in the right area, the 4^th^ leaf stage falls in the left area (with one exception for one *PetE:KNAT1* head stage sample). The control samples show the best separation of two developmental stages. In the case of GM samples, the separation dependence on the growth stage is less evident, suggesting that the growth can be affected by other factors, including the transgene. Finally, PC2 is the most effective in discriminating conventional lettuce from the GM lettuce: by tracing an horizontal line it is possible to separate wild type from the *PetE:KNAT1* genotypes.

**Figure 4:**
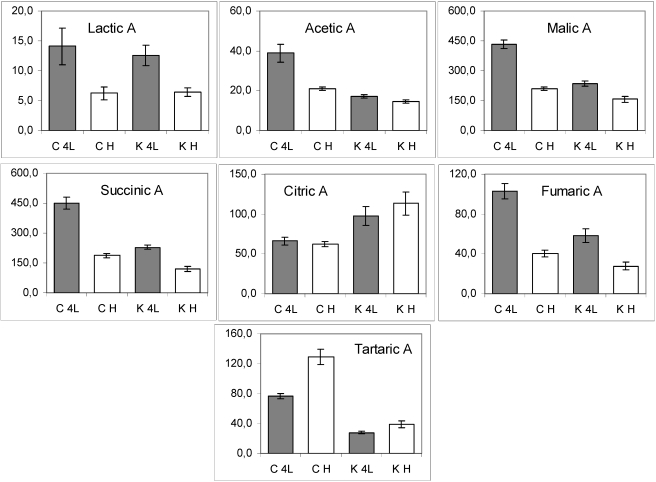
The histograms of molecular abundance (mean values and standard errors) of organic acids in lettuce leaf samples. C, conventional samples; K, *PetE:KNAT1* samples. K4L, GM samples at 4^th^ leaf stage; KH, GM samples at the head stage; C4L, control samples at 4^th^ leaf stage; CH, control samples at the head stage.

**Figure 5:**
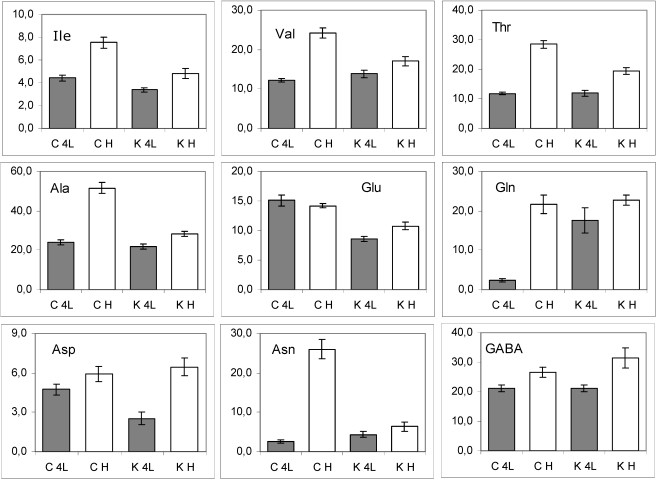
The histograms of molecular abundance (mean values and standard errors) of amino acids in lettuce leaf samples. C, conventional samples; K, *PetE:KNAT1* samples. K4L, GM samples at 4^th^ leaf stage; KH, GM samples at the head stage; C4L, control samples at 4^th^ leaf stage; CH, control samples at the head stage.

**Figure 6:**
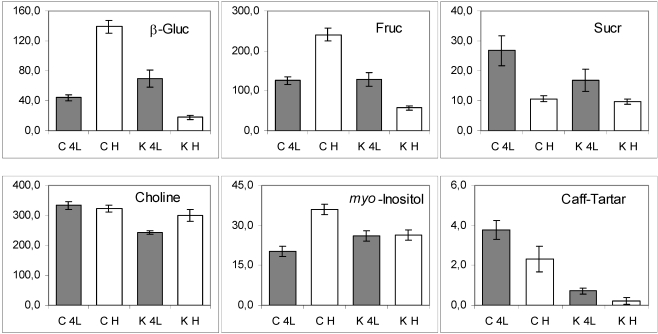
The histograms of molecular abundance (mean values and standard errors) of mono- and disaccharides and other metabolites in lettuce leaf samples. C, conventional samples; K, *PetE:KNAT1* samples. K4L, GM samples at 4^th^ leaf stage; KH, GM samples at the head stage; C4L, control samples at 4^th^ leaf stage; CH, control samples at the head stage.

The influence of each of the two factors, *i.e.* leaf development and genotype, inclusive of the transgene effect, on a single metabolite can be evaluated using a simple 2^2^ factorial design scheme [21] and the histograms of the mean values shown in Figure 4, Figure 5 and Figure 6. In the factorial-design scheme every factor has two levels: the genetic background factor has Control (C) or *PetE:KNAT1* (K) genotype levels, whereas the leaf development factor has 4^th^ leaf (4L) or head stage (H) levels. Four factor combinations, *i.e.*, C-4L, C-H, K-4L and K-H exist. For each combination a suitable number of samples (9-15 replications) was analyzed.

The effect of each factor, *i.e.*, the genotype and the leaf development, on the level of each metabolite can be calculated separately in cases where no interaction between the factors is obvious. The statistically significant increase or decrease of a particular metabolite level due to a specific factor is denoted by + or -, respectively (Table 1). For instance, the significant decrease of Ile content in GM with respect to control samples is denoted by “-“ in Table 1. The occurrence of significant interaction between the two factors can also be determined in the factorial scheme. The interaction, reported in Table 1 with an asterisk, means that the genotype and leaf development can influence each other. For example, as in the case of malic acid, the leaf development modulates or alters the genotype effect and *viceversa*, the transgene expression modulates the leaf maturation.

**Table 1:** Factor analysis of water-soluble metabolites in transgenic and conventional lettuce.

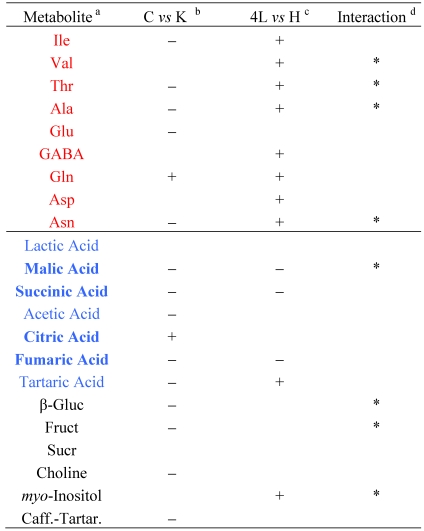

a. In red, amino acids. In blue, organic acids (in bold those of Krebs cycle); in black, mono-and disaccharides and other compounds. b. C, conventional samples; K, *PetE:KNAT1* samples. The increased (+) or decreased (-) level of a metabolite is indicated when found to be so for both the growth stages. c. 4L, samples at 4th leaf stage; H, samples at the head stage. The level variation of a leaf metabolite at head stage and young phase (4L) is indicated when found identical for both transgenic and conventional plants. d. The occurrence of interaction between leaf development and transgene action was assessed for 95% confidence interval.

*Organic acids*. The trend of organic acid content during the growth (4^th^ leaf stage- head stage) was substantially variable in both GM and C genotypes (Figure 4). The decrease of most organic acids was observed in *PetE:KNAT1* leaves with respect to controls, with the exception of citric acid that maintained a higher level in the GM samples during the growth. 

The data in Table 1 are indicative of whether the content of a specific metabolite is a result of the transgene interacting with plant developmental phase. For instance, as previously highlighted with regard to malic acid (Table 1, asterisks; Figure 4, third panel), its content decreased two-fold during the development of control plants, whereas a 1.4-fold decrease was observed in the case of GM line. The statistical analysis indicates that, in the case of malic acid, the effects of genetic background and growth stage are not independent from each other (with 95% confidence). Analyses performed on other independent *Pet:KNAT1* Luxor lines (not shown) suggest that the developmental effects are modulated by that of the transgene rather than by the transformation process. Finally, the independent effect of both factors are clearly seen for tartaric acid (the last panel, Figure 4). 

*Amino acids (AA).* An increase in the AA content was observed during the growth (4^th^ leaf - head stage) of both transgenic and control plants (Figure 5), with the exception of glutamic acid (Glu). The molecular abundances for most of the AA were significantly different in GM relative to control leaves. Moreover, a marked interaction between genotype and development was revealed for Val, Thr, Ala and Asn (asterisked in Table 1). In other words, the level of these AA increased during the leaf development, but the increase was significantly higher in conventional than in *PetE:KNAT1* lettuce. 

*Mono- and disaccharides*. As for β-glucose and fructose, the variation trends at distinct growth stages of conventional genotype were opposite to those of GM line (Figure 6), while the trend for sucrose was less variable (Figure 6, third panel). The level of both mono-saccharides was influenced by the interaction between the transgene and development factors (Table 1). Interestingly, such interaction suggests that the transgene strongly contrasts (rather than moderate) the developmental factor leading to a drop in β-glucose and fructose. This observation suggests that the simple sugars are major, if not direct, targets of *KNAT1* control. 

*Other compounds*. With regard to the caffeoyl-tartaric acid, *PetE:KNAT1* leaves showed a significantly lower content than controls. Caffeic acid and its derivatives are precursors in the biosynthesis of lignin units, hence the diminishment of caffeoyl-tartaric acid suggests that lignin content may also be decreased. This is consistent with the low lignin content of plants overexpressing *KNAT1* [22], which is known to target lignin genes in model species. 

## 3. Experimental Section

### 3.1. Plant Material and Sampling

Sister plants derived from a single mother (*Lactuca sativa* cultivar “Luxor”, compact butter head type, Nunhems, http://www.nunhems.com, out of production) provided leaves for *Agrobacteriumtumefaciens* mediated genetic transformation, which was performed with the empty vector (*pVDH282:00*) and target construct (*pVDH282-PetE:KNAT1*). Primarily transformed lines (T_0_) precociously manifested the phenotype of lobed leaves, caused by *KNAT1* ectopic expression in simple leafed species [13]. Independent transgenic lines were selected for a single T-DNA locus by southern blot analysis and selfed in controlled conditions to reach T-DNA homozygosis. “Luxor” has a low natural out-crossing rate (0.5%) in glass house high density layout cultivation [14]. Transgenic lines with empty constructs did not differ from the wild type at the phenotypical level, whilst several independent *PetE:KNAT1* lines shared similar altered traits. In this work, plants of the T_6_ progeny (homozygous for one T-DNA) derived from controlled self-pollination of the *PetE:KNAT1*-177 line (mild phenotype) were compared to non transformed Luxor individuals descending from the original mother plant used for transformation. The metabolic analyses were also performed on two other independent GM lines and no significant differences were observed for the target compounds (unpublished data) under the conditions reported here, suggesting that metabolic changes reflected transgene expression rather than transformation processes. 

Seeds of both *PetE:KNAT1* (homozygous for one T-DNA copy, T_6_ generation) and conventional lettuce cv "Luxor" (short day nunhems *etc.*) were sown on April 16^th^. The growth rate of the *PetE:KNAT1* plants is slower than that of the conventional type [23]. The first sampling was performed when the 4^th^ leaf was completely expanded, which occurred from the 3^rd^ to 4^th^ week after sowing (WAS) for controls and from the 5^th^ to 6^th^ WAS for transgenics. The leaflets (n = 4) of three plants formed one pool. Fifteen pools and 11 pools of control and transgenic plants were analyzed, respectively. The second sampling was performed at head stage, which occurred from 11 and 12 WAS for controls and GM lines, respectively. The leaves (n = 6) borne in the middle of rosette of one distinct plant formed one pool. Fourteen and nine pools of control and transgenic lines were collected. The samples were lyophilized according to the procedure reported previously [8] and stored at -20 °C. Seeds of both *PetE:KNAT1* and conventional lettuce cv "Luxor" were germinated in trays (ca. 3 dm^3^ per well) containing soil, peat and sand into 1:1:1 ratio. At the 8^th^ leaf, plants were put into 10^3^ cm^3^ pots and then into 20^3^ cm^3^ from ca. 25 leaves onwards in peat and soil (1:3) plus 2 g of fertilizer containing 12% N (5% NH_4_^+^ and 7% NO_3_^-^) per kilogram of substrate. Plants were grown in the greenhouse under a natural light/dark (16/8 h) cycle at 100 μmol m^-2^ s^-1^ flux of photosynthetically active radiation (PAR), at 25 °C.

### 3.2. NMR Analysis

Water-soluble extracts were prepared as reported previously [6]: 1 mL of a D_2_O phosphate buffer, having 400 mM salt concentration, pD value of 6.5 and containing 0.3 mM of 3-(trimethyl-silyl)propionic-2,2,3,3-*d*_4_ acid sodium salt (TSPA) - 0.05 mM EDTA, was mixed with 21.0 ± 0.2 mg of powdered vegetable tissues. The mixture was centrifuged at 10,000 rpm (5,600 g) for 7 min and the supernatant obtained was filtered and transferred into a standard 5 mm NMR tube.

NMR spectra of the extracts were recorded at 300 K on a Bruker AVANCE AQS600 spectrometer operating at the proton frequency of 600.13 MHz and equipped with a Bruker multinuclear z-gradient inverse probehead capable of producing gradients in the z-direction with a strength of 55.4 G/cm. Proton spectra were referenced to the signals of TSPA methyl group at δ = 0.00 ppm in D_2_O phosphate buffer. The ^1^H spectra of the aqueous extracts were acquired by co-adding 512 transients with a recycle delay of 2.5 s and 32K data points (acquisition time 40 min). The residual HDO signal was suppressed using a presaturation during the relaxation delay with a long single soft pulse. To avoid possible saturation effects, the experiment was carried out by using a 45° flip angle pulse of 8.0 μs. 

2D NMR experiments, namely ^1^H-^1^H TOCSY and ^1^H-^13^C HSQC, were performed using the same experimental conditions as previously reported [6]. 

### 3.3. Measurement of the Metabolic Content in Aqueous Extract

The intensity, *i.e.* the peak height, of 22 ^1^H resonances due to assigned water-soluble metabolites were measured with respect to the intensity of TSPA signal (0.3 mM) used as internal standard and normalized to 1000. The twenty two measured resonances are due to Ile (1.02 ppm), Val (1.05 ppm), lactic acid (1.32 ppm), Thr (1.34 ppm), Ala (1.49 ppm), acetic acid (1.92 ppm), Glu (2.07 ppm), GABA (2.30 ppm), malic acid (2.38 ppm), succinic acid (2.41 ppm), Gln (2.48 ppm), citric acid (2.53 ppm), Asp (2.80 ppm), Asn (2.91 ppm), choline (3.21 ppm), β-glucose (3.24 ppm), *myo*-inositol (3.29 ppm), fructose (4.02 ppm), tartaric acid (4.34), caffeoyl-tartaric acid (5.31 ppm), sucrose (5.42 ppm), and fumaric acid (6.52 ppm)

### 3.4. Statistical Analysis

The statistical treatment of the NMR data was performed using the STATISTICA package for Windows (version 5.1, 1997). Before performing the statistical analysis, all the 22 selected variables were mean-centred and each variable was divided by its standard deviation (autoscaling). Principal component analysis (PCA) was performed using 22 variables. In the tree clustering analysis, the Euclidean distance and the complete linkage method were used to measure the distance between samples in the 22-dimensional space and between clusters, respectively. The effects and interactions reported in the Table 1 were statistically significant within the 95% confidence interval. 

## 4. Conclusions

In the study case of "Luxor" lettuce, NMR metabolic profiling ascertained that the *PetE:KNAT1* and wild type lines shared the same compounds under the described conditions, consistently with the previous reports on transgenic tomato [9], maize [10] and lettuce [8]. The NMR profiling together with proper statistical methods enabled characterizing the variability of a set of water-soluble metabolites associated with both developmental processes and transgene expression. The variations in the metabolite levels derived from the interaction between the growth stage and transgene action were also identified. The most relevant metabolic changes were associated with lettuce leaf development, although the maturation was modulated by the transgene expression. The highest impact of *PetE:KNAT1* with the development was observed in the trend of glucose and fructose contents which was inverted with respect to the wild type, suggesting that the overexpression of the transcription factor directly targets the metabolism of these sugars. Finally, the NMR metabolic patterns provided useful data and novel indications to address biological question on *KNAT1* function and the respective targets during development.
